# Cardiac surgery–associated AKI and the risk of progression to end-stage renal disease. A retrospective cohort study

**DOI:** 10.1080/0886022X.2026.2719277

**Published:** 2026-09-14

**Authors:** Matias Hilska, Tomi Toukola, Jan-Ola Wistbacka, Simo-Pekka Koivisto, Tomi Sarkkinen, Aapo Pikkujämsä

**Affiliations:** ^a^ Department of Anaesthesiology and Intensive Care, Vaasa Central Hospital, The Wellbeing Services County of Ostrobothnia, Vaasa, Finland; ^b^ Department of Anaesthesiology, Intensive Care, Emergency Care and Pain Medicine, Faculty of Medicine, University of Turku, Turku, Finland; ^c^ Department of Nephrology, Vaasa Central Hospital, The Wellbeing Services County of Ostrobothnia, Vaasa, Finland; ^d^ Department of Cardiothoracic Surgery, Oulu University Hospital, Oulu, Finland; ^e^ Research Unit of Translational Medicine, Medical Research Centre, University of Oulu, Oulu, Finland

**Keywords:** End-stage renal disease, acute kidney injury, ESRD, AKI, cardiac surgery–associated acute kidney injury, peri-operative kidney injury

## Abstract

**Introduction:**

Cardiac surgery–associated acute kidney injury (CSA-AKI) is associated with increased post-operative complication rate and decreased survival. There is limited knowledge regarding the long-term risk of progressing to end-stage renal disease (ESRD) following cardiac surgery.

**Methods:**

We analyzed the association of CSA-AKI and the cumulative incidence of progression to ESRD in a cohort of consecutive non-emergent cardiac surgical patients operated between 2000 and 2016. Peri-operative data were collected prospectively, while survival and ESRD data were collected retrospectively. We compared the risk of progression to ESRD in patients who experienced mild or moderate-to-severe CSA-AKI to those who did not. Secondarily, we analyzed survival in these respective groups of patients.

**Results:**

The median survival time was 13.7 years (95% confidence interval [CI] 13.3,14.1), over a median follow-up time of 11.9 years (interquartile range 7.8,16.6) among the 3753 patients included in the study. Mild AKI occurred in 383 (10.2%) patients, and moderate-to-severe AKI in 129 (3.4%) patients. Altogether, 63 patients developed ESRD during follow-up, and the 20-year cumulative incidence of ESRD was 1.8% (CI 1.4%, 2.4%). When compared to patients without AKI, the hazard ratio of progression to ESRD was 2.62 (CI 1.36, 5.06) in patients with mild AKI and 8.56 (CI 4.33, 16.9) in patients with moderate-to-severe AKI. Peri-operative AKI was also associated with worse long-term survival and with more frequent post-operative complications.

**Conclusion:**

A clinically significant proportion of patients progress to ESRD during a long-term follow-up after cardiac surgery. CSA-AKI is associated with an increased risk of progressing to ESRD.

## KEY MESSAGES

What is known: Cardiac surgery–associated AKI (CSA-AKI) is a known perioperative complication associated with worse immediate post-operative outcomes and with worse long-term survival.

This Study add: CSA-AKI – even if resolved during primary hospital stay – is associated with a higher risk of progressing to ESRD years after the incident.

Potential Impact: Patients at risk for subsequent progression to ESRD could be identified after cardiac surgery, supporting more targeted long‑term renal follow‑up.

## GRAPHICAL ABSTRACT

**Figure:**
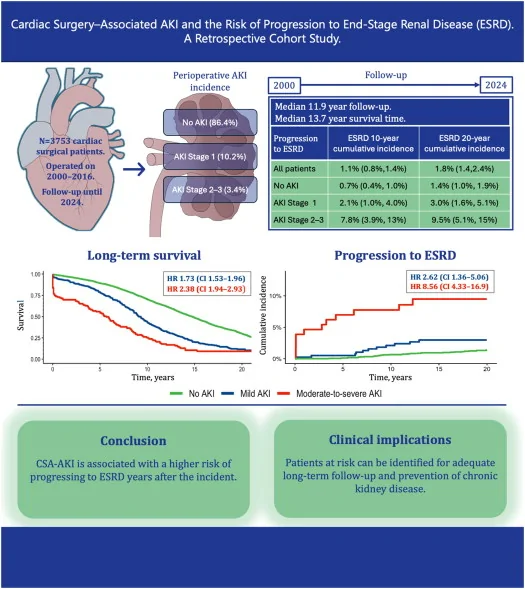

## Introduction

Acute kidney injury (AKI) is defined as a sudden loss of excretory kidney function and is divided into stages 1 to 3 according to severity [1]. Cardiac surgery associated AKI (CSA-AKI) is a well-known peri-operative complication, with an incidence of 22%-27% reported in earlier studies [2–4]. Further, the pooled incidence of CSA-AKI stage 1 is 17.9%, stage 2 is 4.4% and stage 3 is 3.5% [3]. Major risk factors associated with the development of CSA-AKI are pre-operative chronic kidney disease (CKD), decreased systolic heart function, diabetes mellitus, chronic obstructive pulmonary disease and extensive atherosclerotic disease [5,6]. CSA-AKI is associated with increased short- and long-term mortality in studies reporting median follow-up times up to 7 years [2,7–9].

CSA-AKI may also lead to the development of CKD, and further, to end-stage renal disease (ESRD). AKI may cause irreversible damage to the nephrons and facilitate progression to CKD even if the estimated glomerular filtration rate seems to fully recover [10,11]. In one study, the cumulative incidence of ESRD following coronary artery bypass grafting was 0.4% in a mean 4.3 years of follow-up, and it was observed to be higher in patients with CSA-AKI [12].

We aim to examine the association of CSA-AKI and the development of ESRD in Finnish cardiac surgical patients. Secondarily, we report the association of CSA-AKI and long-term survival in our cohort of patients with substantially longer follow-up times than in earlier reports. We hypothesize that patients with CSA-AKI progress to ESRD more frequently than patients whose kidney function was not affected by the surgery.

## Methods

This is a single-centre retrospective cohort study carried out in Vaasa Central Hospital, Finland. The study was retrospectively registered and ethically approved by the institutional review board for medical research in the Wellbeing Services County of Ostrobothnia, Finland (Approval number ÖVPH/1192/13.01/2025). Informed patient consent was waived as the study used routinely collected data and involved no intervention in patient care. The study was conducted in accordance with the Declaration of Helsinki, and the reporting follows the STROBE guidelines [13]. (Supplementary Checklist)

All non-emergent cardiac surgical patients operated during the years 2000 to 2016 at a public secondary level hospital were included in the study. Peri-operative data from all cardiac surgical patients was collected prospectively into an electronic registry until 30 days post-surgery for quality control and research purposes [14,15]. Salvage and emergent surgeries according to Euroscore II criteria were excluded, as well as patients who had ESRD prior to surgery [16]. Surgical and anesthetic technique is described in detail in earlier reports [14,15,17].

We obtained causes and times of death retrospectively from the national population registry, Statistics Finland. We obtained diagnoses and dates of ESRD from the electronic health records of our institution in a comprehensive electronic search complemented with an in-person review (MH) for the following conditions: the construction of arteriovenous fistula, the implantation of a peritoneal dialysis catheter or for the disease codes N18.0 (ESRD), N18.5 (Chronic kidney disease, stage 5) and Z49.1 (Extracorporeal dialysis) in the 10^th^ revision of the International Classification of Diseases. ESRD diagnoses could be retrieved starting from October 2008 when the current electronic health record system was launched in our institution. Follow-up data were collected until January 2024.

The primary outcome measure was the cumulative incidence of ESRD following cardiac surgery. The secondary outcome measure was long-term survival following cardiac surgery. Thirdly, we report early (30-day) post-operative complications.

We calculated the estimated glomerular filtration rates (eGFR) from patient data with the CKD-EPI 2021 formula [18]. KDIGO guidelines were used to classify pre-operative renal function from CKD stages 1 to 5, and peri-operative AKI from stages 1 to 3 [1,19]. For the purposes of the analyses, CKD stages 4 to 5 and AKI stages 2 to 3 were combined due to low numbers of patients. We refer to AKI stage 1 as mild AKI and AKI stages 2 to 3 as moderate-to-severe AKI. For the definition of AKI, only the rise in serum creatinine levels criteria were used and not urine output. Urine output is sensitive to fluctuations during the peri-operative period of cardiac surgery and therefore it was not used as a criterion, consistent with the methodology of most previous reports [3]. The post-operative serum creatinine levels used in the definition of AKI are the highest measured during the post-operative hospital stay, a method adopted from the SWEDEHEART cardiac surgery registry [12]. Resolution of AKI was defined as a decrease of serum creatinine levels to within +27 μmol/l of pre-operative levels at the end of hospital stay. Definitions of peri-operative variables are described in Supplementary Methods.

### Statistical analysis

We conducted statistical analyses in R 4.4.3, employing the *survival* package for survival analysis and *cmprsk* package for competing-risks models [20–22]. Continuous variables are reported as medians with interquartile range. Categorical variables are reported as frequencies (number and percentage). Group characteristics were compared using Kruskal–Wallis tests, chi‑square tests, and Fisher’s exact tests where appropriate.

The cumulative incidence of ESRD, treating death as a competing risk, was analyzed as a primary outcome. A Fine–Gray competing risks regression model was used, and results are reported as subdistribution hazard ratios (HR) with corresponding 95% confidence intervals (CI). In addition, ten- and twenty-year cumulative incidences of ESRD and their corresponding CIs were estimated. Survival data were analyzed with the Kaplan–Meier method and the Cox proportional hazards model, and the results are presented as HRs and CIs. Both time-to-ESRD regression and survival regression analyses were made including Euroscore II risk score as covariate in the models [16]. See Supplementary Methods for further information regarding Euroscore II risk score. Statistical significance was considered at a two-sided alpha of under 0.05.

## Results

A total of 4,028 consecutive individual cardiac surgery patients were identified from the institutional database. We excluded 178 patients with missing survival data, 32 patients with missing kidney function data, 6 patients who had ESRD prior to surgery, and 59 patients with salvage or emergent surgeries. Altogether 3,753 patients undergoing non-emergent cardiac surgery were analyzed and they were followed for a median of 11.9 years (interquartile range 7.8, 16.6; maximum 24.0). Peri-operative characteristics of patients, operations and post-operative complications are presented in Table 1. Only 29 patients (0.8%) had CKD grade 4 to 5 and 592 patients (15.8%) had CKD grade 3 prior to surgery. Peri-operative AKI occurred in 512 patients (13.6%): 383 patients (10.2%) had mild AKI and 129 (3.4%) had moderate-to-severe AKI. Serum creatinine levels returned to pre-operative state in 55% of patients with AKI (*n* = 284 patients) prior to hospital discharge. Temporary renal replacement therapy was used in 52 patients (1.4%) in the intensive care unit post-operatively.

**Table 1.** Peri-operative characteristics in patients with and without cardiac surgery–associated acute kidney injury.

	**No AKI** *N* = 3,241	**Mild AKI** N = 383	**Moderate-to-severe AKI** N = 129	*p*-value
**Pre-operative data**				
Age, years (min, max)	67 (16,90)	72 (40,89)	73 (34,87)	<0.001
Females	918 (28%)	89 (23%)	32 (25%)	0.083
Body mass index, kg/m^2^	27.1 (24.6, 29.9)	28.3 (25.9, 31.2)	28.0 (25.7, 30.8)	<0.001
Euroscore II risk score	1.5 (0.9, 2.7)	2.8 (1.6, 5.1)	4.2 (2.1, 8.0)	<0.001
NYHA status				<0.001
1	173 (7%)	12 (4%)	3 (3%)	
2	819 (33%)	60 (20%)	21 (20%)	
3	1,115 (45%)	137 (47%)	47 (44%)	
4	386 (15%)	85 (29%)	36 (34%)	
Missing data	748	89	22	
Diabetes mellitus	554 (17%)	124 (32%)	43 (33%)	<0.001
Cerebrovascular disease	270 (8%)	55 (14%)	19 (15%)	<0.001
Chronic obstructive pulmonary disease	296 (9%)	46 (12%)	17 (13%)	0.071
Extracardiac atherosclerosis	254 (8%)	61 (16%)	15 (12%)	<0.001
Myocadial infarction within 90 days	638 (20%)	105 (27%)	39 (30%)	<0.001
Left ventricle ejection fraction				<0.001
>50%	2,226 (72%)	220 (61%)	67 (55%)	
31-50%	745 (24%)	116 (32%)	41 (34%)	
21-30%	109 (4%)	20 (6%)	11 (9%)	
<21%	16 (1%)	5 (1%)	3 (3%)	
Missing data	145	22	7	
Glomerular filtration rate, ml/min/1.73 m^2^	82 (68, 93)	67 (53, 83)	62 (42, 78)	<0.001
Anemia	589 (18%)	99 (26%)	47 (36%)	<0.001
**Operative data**				
Urgency of operation				<0.001
Elective	2,590 (80%)	262 (68%)	87 (67%)	
Urgent	651 (20%)	121 (32%)	42 (33%)	
Procedure				0.002
CABG +/- other	2,680 (83%)	344 (90%)	110 (85%)	
Non-CABG	577 (18%)	41 (11%)	20 (15%)	
Euroscore II procedure class				<0.001
Isolated CABG	2,390 (74%)	286 (75%)	76 (59%)	
Single non-CABG	342 (11%)	63 (16%)	39 (30%)	
2 procedures	468 (14%)	33 (8.6%)	14 (11%)	
3 procedures	41 (1%)	1 (0%)	0 (0%)	
Off-pump CABG	418 (13%)	41 (11%)	9 (7%)	0.074
Cardiopulmonary bypass time, minutes	103 (84, 128)	110 (93, 142)	127 (102, 178)	<0.001
Aortic cross-clamp time, minutes	81 (64, 101)	87 (69, 113)	94 (71, 139)	<0.001
Missing data	46	6	4	
Intraoperative allogenic blood products				<0.001
0	2,581 (80%)	255 (67%)	59 (46%)	
1–2	489 (15%)	83 (22%)	31 (24%)	
3–4	113 (4%)	28 (7%)	21 (16%)	
>4	58 (2%)	17 (4%)	18 (14%)	
Intraoperative bleeding, ml	300 (250, 450)	400 (300, 500)	400 (300, 700)	<0.001
Post-operative Hemoglobin, g/l	103 (95, 113)	98 (91, 105)	96 (89, 102)	<0.001
**Post-operative data**				
Hospital stay, days	7 (5, 8)	8 (6, 11)	13 (8, 22)	<0.001
Intensive care unit stay, days	1 (1, 1)	1 (1, 3)	6 (2, 13)	<0.001
Early reoperation	135 (4%)	51 (13%)	34 (26%)	<0.001
30-day mortality	14 (0%)	10 (3%)	20 (16%)	<0.001
Stroke	58 (2%)	24 (6%)	6 (5%)	<0.001
Transient ischemic attack	18 (1%)	1 (0%)	4 (3%)	0.009
Q-wave myocardial infarction	37 (1%)	8 (2%)	8 (6%)	<0.001
Pneumonia	122 (4%)	69 (18%)	45 (35%)	<0.001
Sternal wound infection	20 (1%)	18 (5%)	5 (4%)	<0.001
Delirium	286 (9%)	83 (22%)	41 (32%)	<0.001
Atrial fibrillation	1,239 (41%)	210 (59%)	91 (78%)	<0.001
Missing data	185	30	12	
Renal replacement therapy	0 (0%)	0 (0%)	52 (40%)	<0.001
Intra-aortic balloon pump use	20 (1%)	6 (2%)	17 (13%)	<0.001
Maximum serum creatinine, μmol/l	84 (72, 97)	143 (122, 164)	262 (202, 371)	<0.001

Continuous variables are presented as medians with interquartile ranges in parentheses, except for age, which is presented as mean with minimum and maximum values in parentheses. Categorical variables are presented as counts and within-group percentages.

Percentages may not total 100% due to rounding. Missing data counts exceeding 5 observations are reported.

Urgent operation is defined as a patient not electively admitted who requires surgery during the same hospitalization for medical reasons.

*P*-values are derived from Kruskal-Wallis rank sum test for continuous variables and Pearson’s Chi-squared test or Fisher’s exact test for categorical variables.

Abbreviations: AKI = Acute Kidney Injury, NYHA = New York Heart Association functional status, CABG = Coronary artery bypass grafting.

Pre-operative comorbidities including diabetes mellitus, cerebrovascular disease, chronic obstructive pulmonary disease, extracardiac atherosclerosis, history of myocardial infarction within 90 days prior to surgery, and anemia were all associated with AKI, as well as age, body mass index, Euroscore II, New York Heart Association (NYHA) functional status, and ejection fraction of the left ventricle (Table 1).

AKI occurred more often in urgent than in elective procedures. Cardiopulmonary bypass time and aortic cross-clamp times were longer in patients who developed AKI. AKI was also associated with more intraoperative bleeding, more intraoperative allogenic blood products used and lower post-operative hemoglobin value (Table 1).

Patients with AKI had longer lengths of hospital and intensive care unit stay, and AKI was associated with all the recorded 30-day post-operative complications (Table 1).

### ESRD

**Figure 1.**
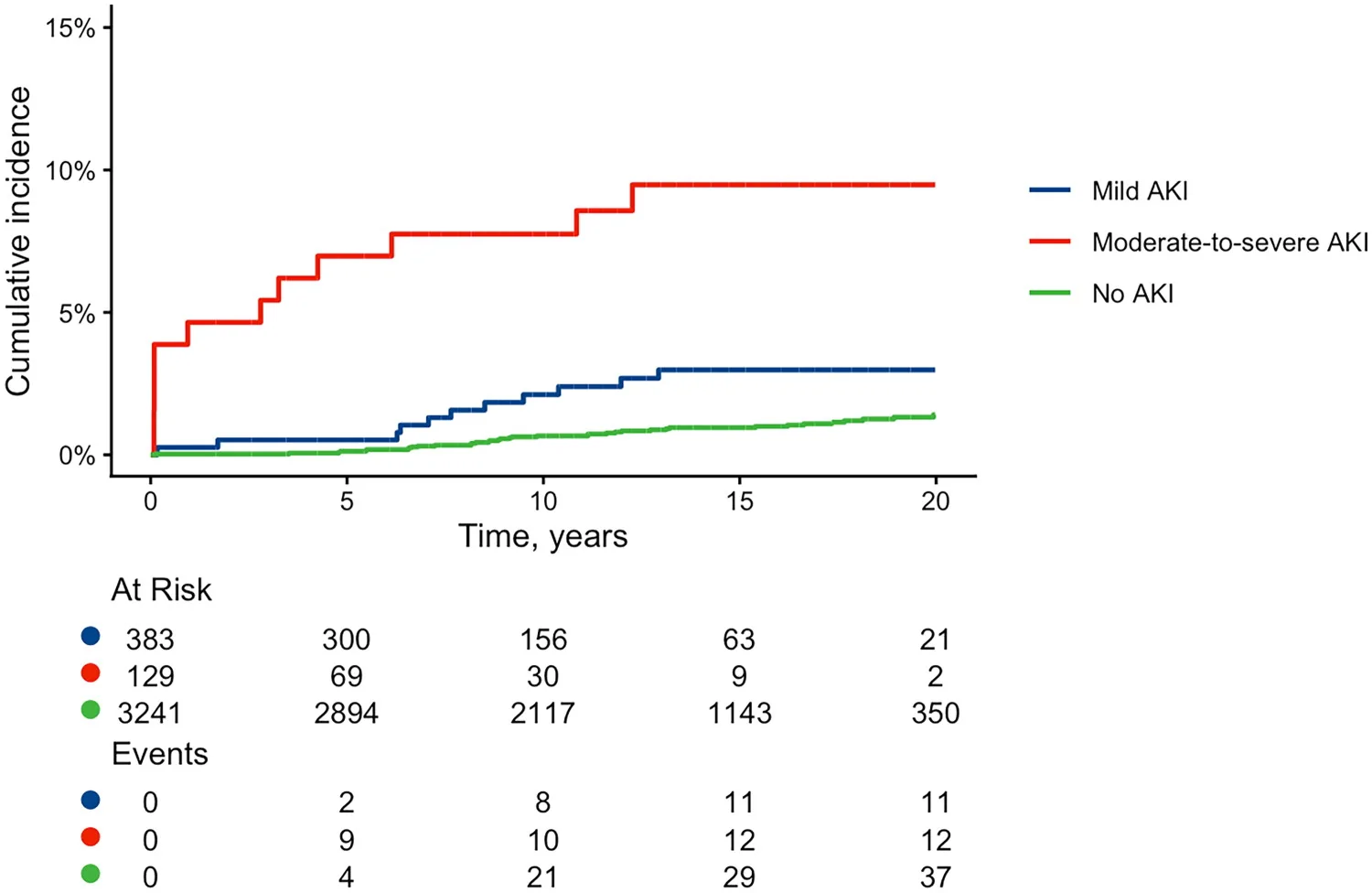
Cumulative incidence curves show the percentage of patients progressing to end‑stage renal disease across CSA-AKI severity groups. The number of patients at risk at each 5‑year interval and the cumulative number of end-stage renal disease events are shown in the risk table beneath the plot. Patients without CSA‑AKI at baseline are shown in green, those with mild AKI in blue, and those with moderate‑to‑severe AKI in red. Fine–Gray regression yielded hazard ratios of 2.62 (CI 1.36, 5.06) for mild CSA-AKI, and 8.56 (CI 4.33, 16.9) for moderate‑to‑severe CSA-AKI, using the no-AKI group as the reference Abbreviations: CSA‑AKI = Cardiac surgery–associated acute kidney injury; CI = 95% confidence interval.

A total of 63 patients progressed to ESRD. The overall cumulative 20-year incidence of ESRD was 1.8% (CI 1.4%, 2.4%), or 9.5% (CI 5.1%, 15%) in patients with moderate-to-severe AKI, 3.0% (1.6%, 5.1%) in patients with mild AKI, and 1.4% (0.99%, 1.9%) in patients without AKI (Figure 1, Table 2). Progression to ESRD was more common in patients with moderate-to-severe AKI, HR 8.56 (CI 4.33, 16.9), as well as with mild AKI, HR 2.62 (CI 1.36, 5.06), when compared to patients without AKI (Table 2).

**Table 2.** Association of the severity of cardiac surgery–associated acute kidney injury and progression to end-stage renal disease.

	10-year cumulative incidence	20-year cumulative incidence
All patients	1.1% (0.8%, 1.4%)	1.8% (1.4, 2.4%)
No AKI	0.7% (0.4%, 1.0%)	1.4% (1.0%, 1.9%)
Mild AKI	2.1% (1.0%, 4.0%)	3.0% (1.6%, 5.1%)
Moderate-to-severe AKI	7.8% (3.9%, 13%)	9.5% (5.1%, 15.0%)

The values represent cumulative‑incidence estimates in percentages at fixed time points, with corresponding 95% confidence intervals in parentheses.

Gray’s test *p*-value <0.001 for difference between No AKI, Mild AKI and Moderate-to-severe AKI groups.

Abbreviations: AKI = Acute kidney injury.

**Figure 2.**
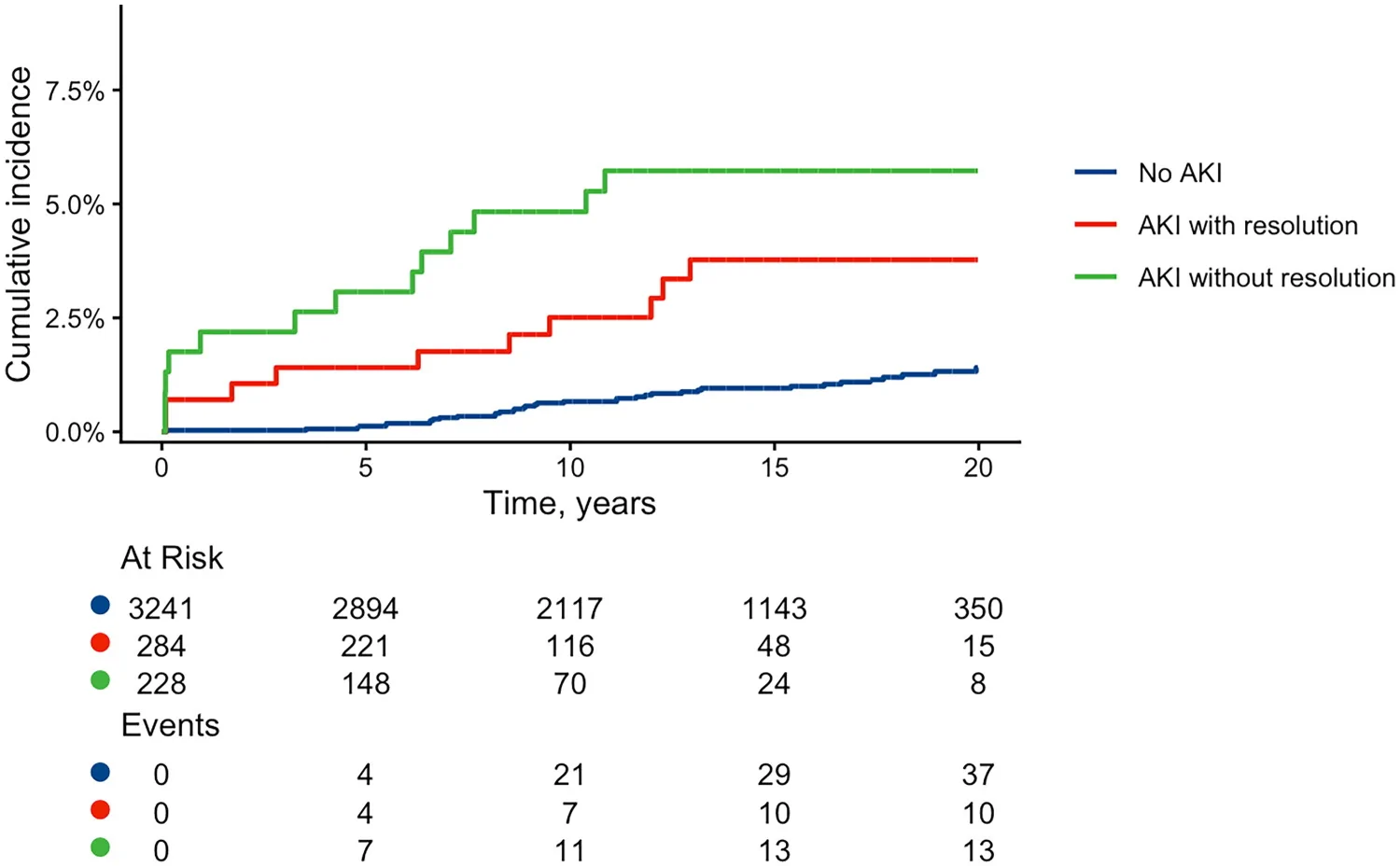
Cumulative incidence curves show the percentage of patients progressing to end‑stage renal disease, stratified by the resolution status of CSA–AKI. The number of patients at risk at each 5‑year interval and the cumulative number of end-stage renal disease events are shown in the risk table beneath the plot. Patients without CSA–AKI at baseline are shown in blue, those with AKI with resolution in red, and those with AKI without resolution in green. Fine–Gray regression yielded hazard ratios of 3.22 (CI 1.62, 6.41) for patients with resolution of AKI, and 4.68 (CI 2.47, 8.89) for patients without resolution of AKI, using the no-AKI group as the reference. Abbreviations: CSA‑AKI = Cardiac surgery‑associated acute kidney injury; CI = 95% confidence interval.

CSA-AKI both with and without resolution during peri-operative hospital stay was associated with higher risk of developing ESRD when compared to patients without AKI: HR 3.22 (CI 1.62, 6.41) for patients with resolution of AKI, and HR 4.68 (CI 2.47, 8.89) for patients without resolution of AKI, in comparison to patients without AKI (Figure 2, Table 3).

**Table 3.** Association of the resolution of cardiac surgery–associated acute kidney injury during peri-operative hospital stay and later progression to end-stage renal disease.

	10-year cumulative incidence	20-year cumulative incidence
No AKI	0.7% (0.4%, 1.0%)	1.4% (1.0%, 1.9%)
AKI with resolution	2.5% (1.1%, 4.9%)	3.8% (1.9%, 6.6%)
AKI without resolution	4.8% (2.6%, 8.2%)	5.7% (3.2%, 9.3%)

The values represent cumulative‑incidence estimates in percentages at fixed time points, with corresponding 95% confidence intervals in parentheses.

Gray’s test *p*-value <0.001 for difference between groups.

Resolution of AKI is defined as a decrease of serum creatinine levels to within +27 μmol/l of pre-operative levels at the end of hospital stay.

A total of 271 patients died before systematic collection of ESRD diagnoses began. See Supplementary Figure 1 and Supplementary Table 1 for a sensitivity analysis of progression to ESRD restricted to patients operated from 2008 to 2016. See Supplementary Figure 2 and Supplementary Table 2 for a sensitivity analysis of progression to ESRD restricted to patients with baseline eGFR ≥ 60.

### Survival

**Figure 3.**
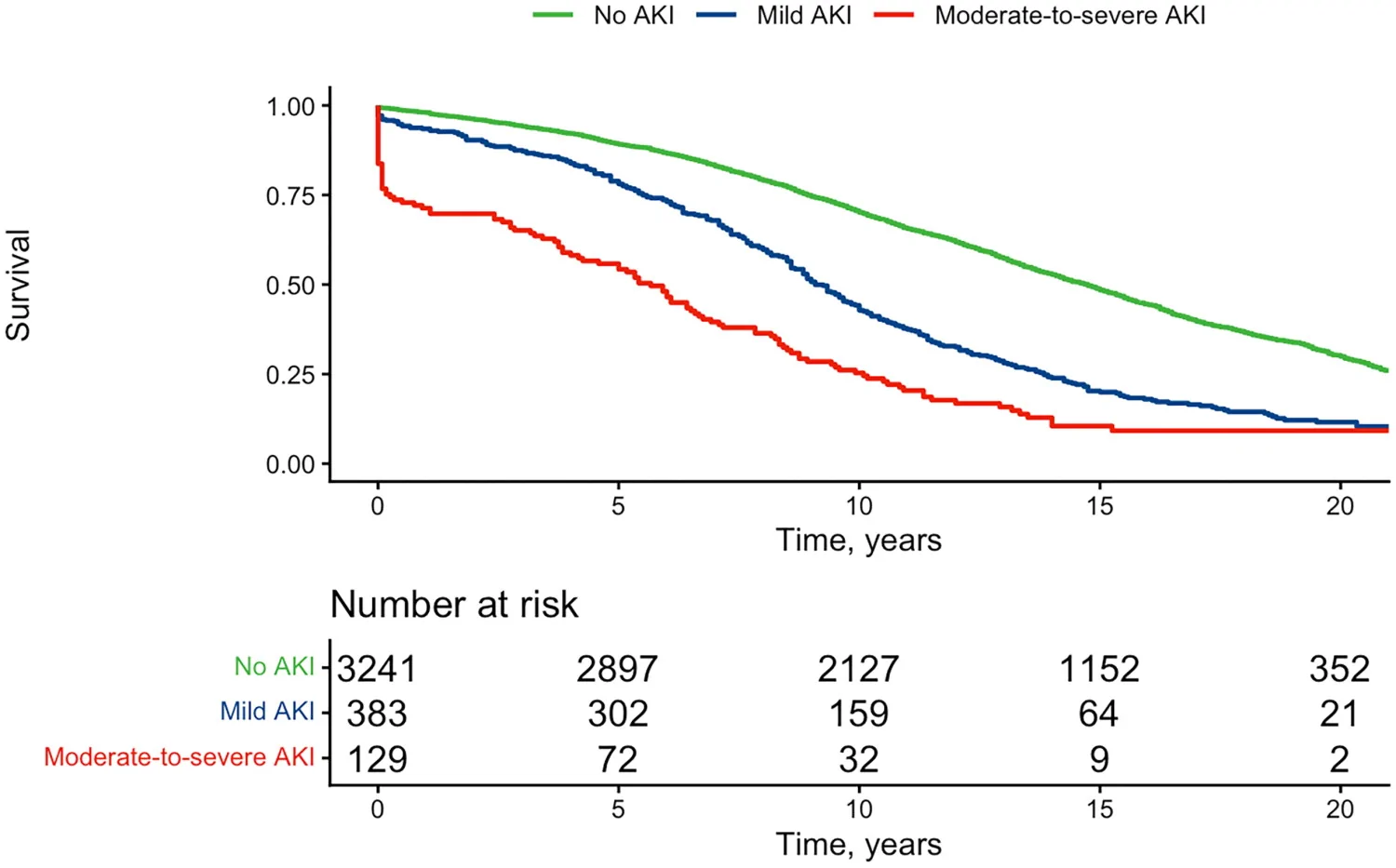
Kaplan–Meier curves depict overall survival, showing the proportion of patients alive at each time point across CSA-AKI severity groups. The number of patients at risk at each 5‑year interval is presented in the risk table beneath the plot. Patients without CSA–AKI at baseline are shown in green, those with mild CSA-AKI in blue, and those with moderate‑to‑severe CSA-AKI in red. Cox proportional hazards regression yielded hazard ratios for death of 1.73 (CI 1.53,1.96) in patients with mild AKI and 2.38 (CI 1.94, 2.93) in patients with moderate-to-severe AKI, using the no-AKI group as the reference. Abbreviations: CSA‑AKI = Cardiac surgery‑associated acute kidney injury; CI = 95% confidence interval.

A total of 2383 (63.5%) patients died during follow-up. Median survival time was 13.7 years (95% CI 13.3, 14.1) in the whole cohort; or 14.8 years (CI 14.3, 15.2) in patients without AKI, 9.16 years (CI 8.7, 9.9) in patients with mild AKI and 5.7 years (CI 4.2, 6.8) in patients with moderate-to-severe AKI (Figure 2). Survival was worse in both mild and moderate-to-severe AKI groups: HR for death was 1.73 (CI 1.53,1.96) in patients with mild AKI and 2.38 (1.94, 2.93) in patients with moderate-to-severe AKI, when compared to patients without AKI (Figure 3).

A total of 202 patients (8.5% of all deaths) had renal disease as either the primary cause of death (17 patients) or as a contributing cause of death (188 patients). The proportion of deaths in which renal disease contributed increased across AKI severity groups, occurring in 7% of patients without AKI, 13% of those with mild AKI, and 24% of those with moderate-to-severe AKI (*p* < 0.001). Cardiovascular disease was the most common primary cause of death, accounting for 52% of deaths among patients without AKI, 54% among those with mild AKI, and 67% among those with moderate-to-severe AKI (*p* = 0.005).

## Discussion

We present that CSA-AKI is associated with the long-term development of ESRD and with worse long-term survival after cardiac surgery. Even in patients with only mild AKI, the risk of developing ESRD was more than two times higher and the median survival time was more than five years shorter when compared to patients without CSA-AKI. Severe AKI presented a substantial risk in relation to both survival and in developing ESRD. Importantly, even CSA-AKI with complete resolution of kidney function during primary hospital stay was associated with later progression to ESRD and should not be overlooked. High-quality studies addressing these findings and providing extended follow-up comparable to ours are currently not available. The findings of this study should inform clinicians to arrange adequate renal follow-up in patients who experience CSA-AKI.

Earlier studies have reported rates of CSA-AKI of 22% to 27%. We report CSA-AKI incidence of 13.6%, which is an underestimate of the true incidence as we excluded emergency surgeries from our analysis, and we did not include urine output criteria in the definition of CSA-AKI. However, it could be argued that urine output more reflects a hemodynamic derangement than true kidney cellular damage in the context of cardiac surgery, and as such, it would be too sensitive as a marker for AKI. We are also concerned that the current definition of kidney injury relies on functional markers rather than on biomarkers that reflect actual cellular damage [4,23,24]. Risk factors for developing CSA-AKI such as comorbidities and cardiopulmonary bypass time are widely understood, and our study further builds up to this evidence [4]. Research has appropriately already focused on specific interventions to prevent CSA-AKI, and this would arguably be the most effective way to prevent the progression of CKD as well [25].

There is very limited evidence of the long-term incidence ESRD following cardiac surgery, and our study is the first one to include patients without coronary artery disease, i.e. isolated aortic and valve operations as well. Ishani and coworkers reported CSA-AKI to be in association with the development and progression of CKD in a 5-year follow-up, but with no information of its progression to ESRD [9]. One previous study has investigated the incidence of ESRD in patients undergoing non-emergent coronary artery bypass grafting [12]. Firstly, we confirm their findings that CSA-AKI is in strong association with later development of ESRD. Secondly, they found 0.4% of patients progressing to ESRD in a mean follow-up of 4.3 years, whereas we report a cumulative incidence of 1.8% in a median follow-up time of 11.9 years. This difference in cumulative incidence reveals the slow progression of ESRD only after many years of the first insult of mild AKI. We could also demonstrate this visually in Figure 1, where the incidence of ESRD starts to accumulate after 6 years of follow-up. This finding highlights the importance of sufficiently long follow-up times in the study and care of patients surviving AKI and at risk of ESRD.

A recent consensus report guides patients with severe CSA-AKI for follow-up at a nephrologist, but our findings would prompt for the importance of long-term clinical renal follow-up of all CSA-AKI patients [23]. For now, it is not known whether follow-up with a nephrologist after all-cause AKI could reduce progression to ESRD, but mortality benefits have already been reported [26]. Given the recent progress in diagnostics, therapies, and clinical outcomes for CKD patients, follow‑up and interventions should be a subject of further research [27]. Timely primary and secondary preventive interventions could slow down or halt the progress of CKD and result in reduced morbidity, mortality and health-care related costs.

We confirm the findings of Rydén and coworkers that CSA-AKI is in strong association with later development of ESRD. However, these studies do not answer the question whether CSA-AKI is independently the cause for progression to ESRD or merely a surrogate marker of the general critical and vasculopathic status of the patient and their cardiorenovascular system at baseline.

### Strengths and limitations

Our study includes a high-quality prospective institutional cardiac surgery registry with comprehensive clinical peri-operative data. There were no missing data on peri-operative renal function except for 32 patients who died within hospital stay. Follow-up data could be retrieved from almost everyone in the registry and we present a substantially longer follow-up period than in earlier reports.

A limitation of the study was the retrospective nature of the study. ESRD diagnoses were retrieved from clinical patient archives and there is a possibility that these data are not complete. However, the continuity of care in a Nordic country is very high and all ESRD patients in our area are treated in the same public nephrology clinic. There are no possibilities for private or alternative providers of ESRD care in Finland, so it is highly likely that we could include all relevant patients for this study. An exception to this is that we were unable to include those patients who progressed to ESRD but died prior to 2008; however, this number is likely very small. In addition, these results should be interpreted in the context that many of the operations were performed in the early 2000s, and therefore the findings may not fully reflect modern surgical, anesthetic, or renoprotective practices. We have already addressed the limitation arising from the absence of urine output criteria in the definition of AKI. In addition, information on pre-operative and post-operative albuminuria could have further refined the assessment of CSA-AKI and long-term ESRD risk. Finally, the cumulative incidence of ESRD at the 20‑year time point should be interpreted with caution, as the median follow‑up duration in our cohort was only 11.9 years.

## Conclusion

CSA-AKI is associated with the risk of developing ESRD years after surgery. It is also associated with significantly worse long-term survival when compared to patients without CSA-AKI. It would be wise to arrange adequate screening, follow-up and prevention of CKD in patients with CSA-AKI.

## Supplementary Material

Submit Supplementary Methods Not highlighted.doc

## Data Availability

The data that support the findings of this study are available from the corresponding author upon reasonable request. Restrictions apply to the availability of these data, which were used under license for this study. Data are only available with the permission of the Finnish Social and Health Data Permit Authority Findata. Requesting parties are responsible for all costs related to secure data handling.
