# Supplementary material for: Cardiac surgery–associated AKI and the risk of progression to end-stage renal disease. A retrospective cohort study
Source: Ren Fail. 2026 Sep 14;48(1):2719277. doi: 10.1080/0886022X.2026.2719277 (PMC13577249; doi:10.1080/0886022X.2026.2719277)
Supplement: Supplementary file 1 [file IRNF_A_2719277_SM8226.doc]

# Supplementary Methods

**Euroscore II risk score**

The European System for Cardiac Operative Risk Evaluation II (Euroscore II) is a cardiac risk model for predicting mortality following cardiac surgery with information from preoperative factors. For reference, see: Nashef SAM, Roques F, Sharples LD, et al. EuroSCORE II. *Eur J Cardiothorac Surg*. 2012;41(4):734-745. doi:10.1093/ejcts/ezs043.

The risk score includes age as a continuous variable and 17 categorical variables divided into patient-related factors, cardiac-related factors and operation-related factors. The score represents the risk of dying within 30 days after surgery, expressed as a percentage.

The following variables are included in the Euroscore II risk score:

Patient-related factors: age, sex, chronic lung disease, extracardiac arteriopathy, poor mobility, previous cardiac surgery, active endocarditis, critical preoperative state, diabetes on insulin treatment, and renal impairment. Renal impairment is divided into four classes according to glomerular filtration rate (normal >85 ml/min, moderate 50-85 ml/min, severe <50 ml/min) or on dialysis treatment.

Cardiac-related factors: Canadian Cardiovascular Society’s angina grade, New York Heart Association functional status class, left ventricular ejection fraction (categorised into four classes), history of myocardial infarction within 90 days prior to operation, pulmonary hypertension.

Operation-related factors: surgery on thoracic aorta, urgency of operation, weight of the operation divided into four classes: isolated coronary artery bypass grafting (CABG), single non-CABG, 2 procedures, 3 procedures.

**Other definitions**

Anemia is defined according to the World Health Organization as blood hemoglobin level of under 130 g/l in men and under 120 g/l in women.

Post-operative complications were assessed prospectively, and the diagnoses were made according to current clinical practice. Neurologist was consulted for the diagnosis of stroke and transient ischemic attack. Pneumonia was diagnosed by an intensive care physician or a physician in the cardiac surgical ward. Postoperative myocardial infarction was diagnosed following clinical suspicion with simultaneous new Q-wave in the electrocardiogram (ECG). Sternal wound infection was diagnosed by a cardiac surgeon.

Delirium was diagnosed by an intensive care physician. Atrial fibrillation was diagnosed as an episode of ECG-documented atrial fibrillation, irrespective of its duration.

# Supplementary Checklist – STROBE Statement

|  | Item No | Page number | | Recommendation |
| --- | --- | --- | --- | --- |
| **Title and abstract** | 1 | 1 | | (a) Indicate the study’s design with a commonly used term in the title or the abstract |
|  |  | 4–5 | | (b) Provide in the abstract an informative and balanced summary of what was done and what was found |
|  | | | Introduction | |
| Background/rationale | 2 | 6 | | Explain the scientific background and rationale for the investigation being reported |
| Objectives | 3 | 6 | | State specific objectives, including any prespecified hypotheses |
|  | | | Methods | |
| Study design | 4 | 7 | | Present key elements of study design early in the paper |
| Setting | 5 | 7 | | Describe the setting, locations, and relevant dates, including periods of recruitment, exposure, follow-up, and data collection |
| Participants | 6 | 7 | | (a) Cohort study—Give the eligibility criteria, and the sources and methods of selection of participants. Describe methods of follow-up  Case-control study—Give the eligibility criteria, and the sources and methods of case ascertainment and control selection. Give the rationale for the choice of cases and controls  Cross-sectional study—Give the eligibility criteria, and the sources and methods of selection of participants |
|  |  |  | | (b) Cohort study—For matched studies, give matching criteria and number of exposed and unexposed  Case-control study—For matched studies, give matching criteria and the number of controls per case |
| Variables | 7 | 7–9, suppl. | | Clearly define all outcomes, exposures, predictors, potential confounders, and effect modifiers. Give diagnostic criteria, if applicable |
| Data sources/ measurement | 8 | 7–9,  suppl. | | For each variable of interest, give sources of data and details of methods of assessment (measurement). Describe comparability of assessment methods if there is more than one group |
| Bias | 9 | 7–9 | | Describe any efforts to address potential sources of bias |
| Study size | 10 | 7–9 | | Explain how the study size was arrived at |
| Quantitative variables | 11 | Table 1,  suppl. | | Explain how quantitative variables were handled in the analyses. If applicable, describe which groupings were chosen and why |
| Statistical methods | 12 | 7–9 | | (a) Describe all statistical methods, including those used to control for confounding |
|  |  | 7–9 | | (b) Describe any methods used to examine subgroups and interactions |
|  |  | 8, Table 1 | | (c) Explain how missing data were addressed |
|  |  | 9 | | (d) Cohort study—If applicable, explain how loss to follow-up was addressed  Case-control study—If applicable, explain how matching of cases and controls was addressed  Cross-sectional study—If applicable, describe analytical methods taking account of sampling strategy |
|  |  | 9, 11 | | (e) Describe any sensitivity analyses |

|  | Item No | | Page No | | | Recommendation |  |  |  |
| --- | --- | --- | --- | --- | --- | --- | --- | --- | --- |
|  | | | | | Results | | | |  |
| Participants | | 13 | 9 | | (a) Report numbers of individuals at each stage of study—eg numbers potentially eligible, examined for eligibility, confirmed eligible, included in the study, completing follow-up, and analysed | | | | |
|  |  |  | 9 | (b) Give reasons for non-participation at each stage | | | | |  |
|  |  |  | - | (c) Consider use of a flow diagram | | | | |  |
| Descriptive data | | 14 | Table 1 | (a) Give characteristics of study participants (eg demographic, clinical, social) and information on exposures and potential confounders | | | | |  |
|  |  |  | Table 1 | (b) Indicate number of participants with missing data for each variable of interest | | | | |  |
|  |  |  | 9–10 | (c) Cohort study—Summarise follow-up time (eg, average and total amount) | | | | |  |
| Outcome data | | 15 | 10 | Cohort study—Report numbers of outcome events or summary measures over time | | | | |  |
|  |  |  | - | Case-control study—Report numbers in each exposure category, or summary measures of exposure | | | | |  |
|  |  |  | - | Cross-sectional study—Report numbers of outcome events or summary measures | | | | |  |
| Main results | | 16 | Table 2 & 3 | (a) Give unadjusted estimates and, if applicable, confounder-adjusted estimates and their precision (eg, 95% confidence interval). Make clear which confounders were adjusted for and why they were included | | | | |  |
|  |  |  | Table 1 | (b) Report category boundaries when continuous variables were categorized | | | | |  |
|  |  |  | Table 2 & 3 | (c) If relevant, consider translating estimates of relative risk into absolute risk for a meaningful time period | | | | |  |
| Other analyses | | 17 | 9–11 | Report other analyses done—eg analyses of subgroups and interactions, and sensitivity analyses | | | | |  |
|  | | | | Discussion | | | | |  |
| Key results | | 18 | 11-12 | Summarise key results with reference to study objectives | | | | |  |
| Limitations | | 19 | 13-14 | Discuss limitations of the study, taking into account sources of potential bias or imprecision. Discuss both direction and magnitude of any potential bias | | | | |  |
| Interpretation | | 20 | 14 | Give a cautious overall interpretation of results considering objectives, limitations, multiplicity of analyses, results from similar studies, and other relevant evidence | | | | |  |
| Generalisability | | 21 | 11-14 | Discuss the generalisability (external validity) of the study results | | | | |  |
|  | | | | Other information | | | | |  |
| Funding | | 22 | 17 | Give the source of funding and the role of the funders for the present study and, if applicable, for the original study on which the present article is based | | | | |  |

Reference: von Elm E, Altman DG, Egger M, et al. The Strengthening the Reporting of Observational Studies in Epidemiology (STROBE) statement: guidelines for reporting observational studies. Lancet. 2007;370(9596):1453-1457. doi:10.1016/S0140-6736(07)61602-X
